# Paradoxical risk of reduced fertility after exposure of prepubertal mice to vincristine or cyclophosphamide at low gonadotoxic doses in humans

**DOI:** 10.1038/s41598-020-74862-8

**Published:** 2020-10-20

**Authors:** Marion Delessard, Justine Saulnier, Ludovic Dumont, Aurélie Rives-Feraille, Nathalie Rives, Christine Rondanino

**Affiliations:** Department of Reproductive Biology–CECOS, EA 4308 “Gametogenesis and Gamete Quality”, Rouen University Hospital, Normandie Univ, UNIROUEN, 76000 Rouen, France

**Keywords:** Paediatric cancer, Quality of life, Reproductive disorders

## Abstract

Cancer treatment can have long-term side effects in cured patients and infertility is one of them. Given the urgency of diagnosis in children with cancer, the toxicity of treatments on the gonad was overshadowed for a long time. In the present study, prepubertal mice were treated by vincristine or cyclophosphamide commonly used in acute leukaemia treatment. The prepubertal exposure to cyclophosphamide, at a low gonadotoxic dose in humans (< 3.5 g/m^2^), led to morphological alterations of prepubertal testicular tissue. An increased proportion of spermatozoa with hypocondensed chromatin and oxidized DNA associated with decreased fertility were uncovered at adulthood. Short- and long-term morphological alterations of the testicular tissue, disturbed progression of spermatogenesis along with increased proportions of isolated flagella and spermatozoa with fragmented DNA were evidenced in vincristine-treated mice. Moreover, the fertility of mice exposed to vincristine was severely affected despite being considered low-risk for fertility in humans. Paternal exposure to vincristine or cyclophosphamide before puberty had no impact on offspring development. Contrary to the current gonadotoxic risk classification, our results using a mouse model show that vincristine and cyclophosphamide (< 3.5 g/m^2^) present a high gonadotoxic risk when administered before the initiation of spermatogenesis.

## Introduction

Over the last decades, the long-term survival rate of children with cancer has improved significantly thanks to earlier detection of cancer and progress in treatments^1^. However, cancers remain the second most frequent cause of death for children under 15 years of age. Among childhood cancers, leukaemia (mainly acute lymphoblastic leukaemia) is the most common^2^. The chemotherapy treatment of acute leukaemia includes three main phases: (1) an induction phase, (2) a consolidation phase whose aim is to destroy all tumour cells in the blood and bone marrow and (3) a maintenance chemotherapy to prolong remission in patients^3^. According to several clinical studies, the conventional chemotherapy is presumed with low risk for fertility^4–6^. If the treatment is not efficient, the patients receive hematopoietic stem cell transplantation (HSCT) which first requires an intensive chemotherapy known for its high gonadotoxicity. To prevent side-effects on gonads, procedures of fertility preservation are proposed. In prepubertal boys unable to produce sperm, fertility preservation can be considered using testicular tissue freezing, which remains an experimental procedure^7^. Currently, this procedure is proposed to children with leukaemia exclusively before the high-dose conditioning chemotherapy regimens that precede HSCT. However, it is reasonable to assume that chemotherapy received during the induction and consolidation phases could have adverse effects on the prepubertal testis. Paediatric leukaemia survivors who have not benefited from fertility preservation procedures could present long-term reproductive sequelae, especially if alkylating agents were included in their regimen^5,6,8^. Indeed, a growing number of studies have reported fertility impairment at adulthood after prepubertal exposure to chemotherapy, with a prevalence of male infertility reaching 46% in childhood cancer survivors compared to 17.5% in siblings^9,10^. A classification of the chemotherapeutic agents according to the risks of infertility after exposure in childhood or adulthood has been defined to help clinicians to better refer patients for fertility preservation^11,12^. Since chemotherapy drugs are usually administered in combination, this classification remains questionable as it appears to be difficult to identify the risk of each agent. Moreover, very few data are still available on how these chemotherapeutic agents affect the prepubertal testis and most of the current data are deduced from adult studies^13^. Since the hormonal environment and the germ and somatic cell contents are different between the prepubertal and the adult testis, further studies investigating the impact of chemotherapy exposure before puberty on male fertility are needed^14^.

Cyclophosphamide (CYP) and vincristine (VCR) are classically used in the treatment of acute leukaemia during the induction and consolidation phases. The alkylating metabolite of CYP, phosphoramide mustard, forms DNA adducts and inhibits DNA transcription and replication^15^. It is widely believed that alkylating agents such as CYP present a high risk for fertility, which increases in a dose-dependent manner^11,16^. Human studies have shown that the administration of alkylating agents before puberty results in a depletion of the spermatogonial pool at adulthood compared to therapy without alkylating agents^17,18^. Similarly, a loss of spermatogonial stem cells (SSC) with a significant increase of DNA damage (DNA double-strand breaks) has been found after 24 h and 48 h of in vitro exposure of prepubertal mouse testes to CYP^19,20^. VCR is a vinca-alkaloid that blocks cell division by inhibiting microtubule polymerization^21^. This chemotherapeutic agent is considered to have a low gonadotoxic potential, although studies in the rodent model have shown that an in vitro exposure of spermatogonial cell lines with SSC characteristics to VCR for 48 h resulted in increased apoptosis^11,22^.

In the present study, we assessed for the first time the impact of in vivo exposure of prepubertal mice to VCR or CYP before the initiation of the first wave of spermatogenesis on the testicular tissue, sperm production and quality, fertility and offspring development. VCR (200 µg/kg) and CYP (90 mg/kg) were administered individually to 3-day *postpartum* (d*pp*) mice, age at which the only germ cells present in the testis are gonocytes and spermatogonia^23^. These doses are similar to the low concentrations used in induction and consolidation chemotherapy in the clinics and therefore considered to be at low risk for fertility. The effects of each drug were analysed in the short-term (before entry into meiosis, i.e. 3 days after treatment) and the long-term (after a few cycles of spermatogenesis).

## Results

### Short- and long-term testicular tissue alteration after VCR but not CYP prepubertal exposure

Prepubertal 3-day-old mice received either an intraperitoneal injection of saline solution (NaCl 0.9% group), cyclophosphamide (CYP group) or vincristine (VCR group). Mice in the untreated group did not receive any injection. VCR and CYP groups were compared to the control NaCl 0.9% group throughout this study. Our data revealed no significant difference between the NaCl 0.9% group and the untreated group (Fig. S1).

**Figure 1:**
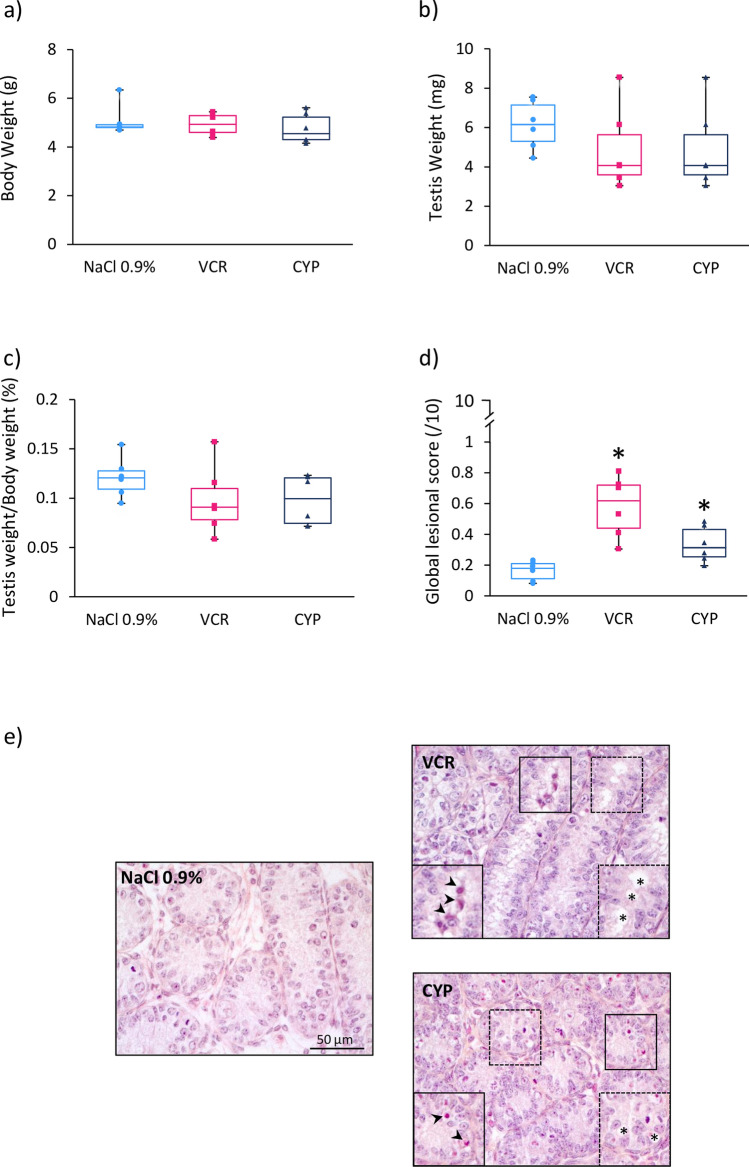
Short-term impact of CYP or VCR exposure on prepubertal testes. Box plots representing **(a**) median body weight, (**b**) median testis weight and (**c**) median ratio of testicular weight to body weight of 6-day-old (D6) mice treated with NaCl 0.9%, VCR or CYP at 3 days of age. Morphological alterations of the seminiferous tubules were assessed semi-quantitatively by calculating a global lesional score (**d**). Representative microscopy images of HES-stained D6 mouse testicular tissues are shown at a × 400 magnification (**e**). Panels on the lower left corner and lower right corner represent enlarged views of pyknotic nuclei (black arrows) and vacuoles (asterisks). Median is indicated by an horizontal line and points represent individual data with n = 6 mice for NaCl 0.9%, VCR and CYP groups. *P < 0.05 compared to NaCl 0.9% group at the same age.

Three days post-treatment (at D6), similar body weights, testis weights and ratios of testis weight to body weight were observed in the different groups (Fig. 1a–c). However, the structural integrity of testicular tissues from VCR (0.63 vs 0.19; *P* < 0.0001) and CYP-treated mice (0.33 vs 0.19; *P* = 0.019) was affected, with a higher global lesional score relative to NaCl 0.9% group (Fig. 1d). The global lesional score (0–10) allowed a semi-quantitative evaluation of the integrity and structural modifications of the testicular tissue including a score between 0 and 5 for nuclear and from 0 to 5 for epithelial alterations (0 representing the complete absence of alteration and 5 representing the most severe alterations). At D6, epithelial and nuclear alterations were noticed, including the presence of vacuoles and pyknotic nuclei in the seminiferous tubules in VCR and CYP groups (Fig. 1e).

**Figure 2:**
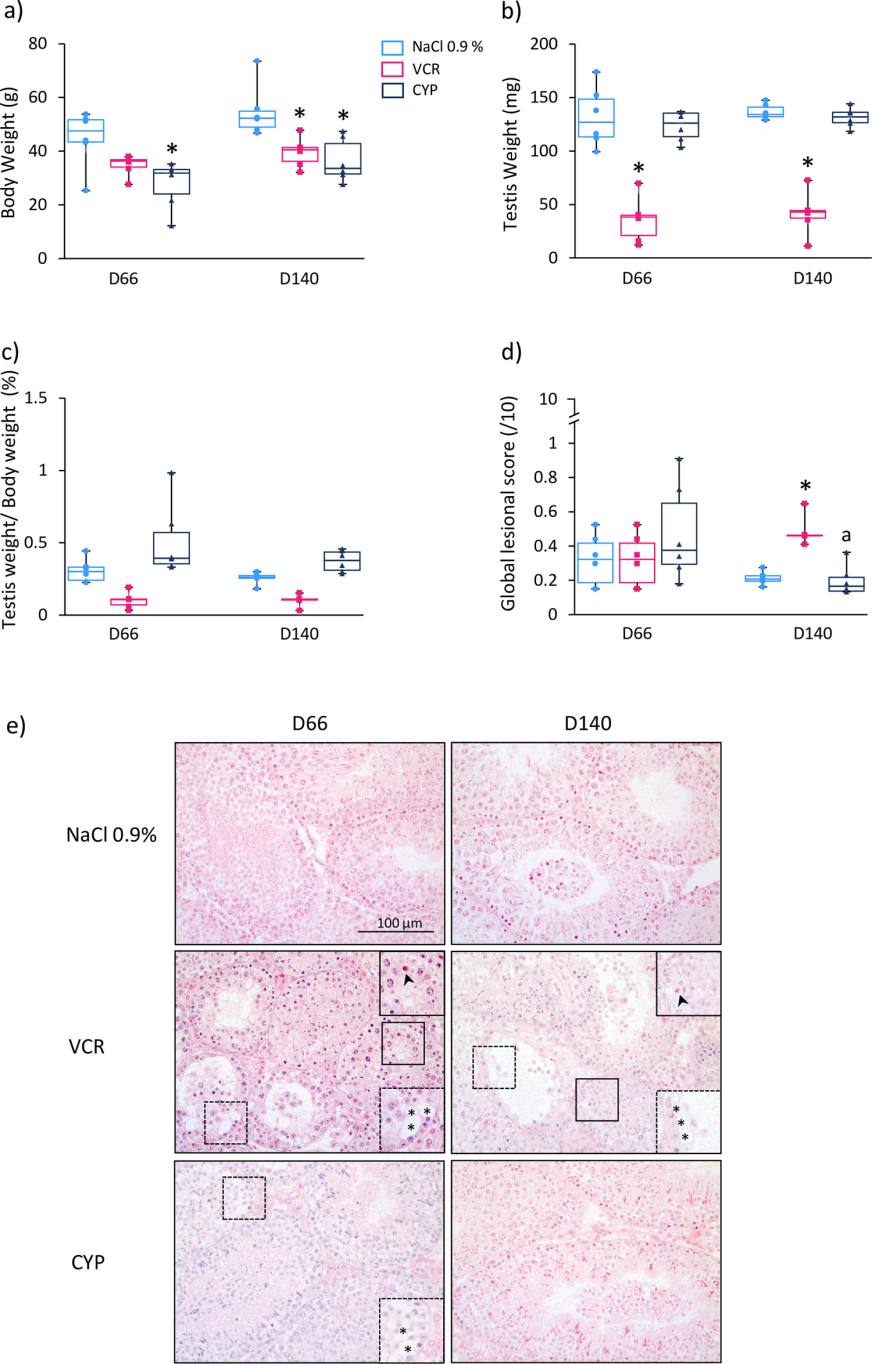
Long-term impact of CYP or VCR prepubertal exposure on D66 and D140 mouse testes. Box plots representing (**a**) median body weight, (**b**) median testis weight and (**c**) median ratio of testicular weight to body weight of D66 and D140 mice treated by NaCl 0.9%, VCR or CYP at 3 days of age. Morphological alterations of the seminiferous tubules were assessed semi-quantitatively by calculating a global lesional score (**d**). Microscopy images of HES-stained testicular tissues from D66 and D140 mice are shown at a × 200 magnification (**e**). Vacuoles are represented by asterisks (*) (enlarged view in the lower right corner) and the presence of pyknotic nuclei is indicated by black arrows (enlarged view in the upper right corner). Median is indicated by an horizontal line and points represent individual data with n = 6 mice for NaCl 0.9%, VCR and CYP groups. *P < 0.05 compared to NaCl 0.9% group at the same age. ^a^*P* < 0.05 compared to CYP group at D66.

A long-term impact on body weight was observed in mice treated with chemotherapy: a decreased body weight was observed in CYP-treated-mice at D66 (31.86 vs 47.55; *P* = 0.0044) and D140 (33.53 vs 52.28; *P* = 0.0038) and in VCR-treated mice at D140 (40.50 vs 52.28; *P* = 0.0112) compared to the NaCl 0.9% group (Fig. 2a). Moreover, males treated by VCR displayed a decreased testis weight at D66 (38.23 vs 126.95; *P* = 0.0058) and D140 (42.96 vs 134.25; *P* = 0.0013) relative to NaCl 0.9% group (Fig. 2b). However, similar ratios of testis weight to body weight were observed in the different groups (Fig. 2c). Despite a 2.7 and 2.4-fold decrease in the ratios of testis weight to body weight respectively, at D66 (0.11 vs 0.30; *P* = 0.0915) and D140 (0.11 vs 0.26; *P* = 0.112) in mice exposed to VCR compared to the NaCl 0.9% group, no significant difference was found (Fig. 2c). No evolution of this ratio was observed in VCR-treated mice between D6 (0.09), D66 (0.11) and D140 (0.11) whereas an increase of at least twofold was observed between D6 and adult age (D66 or D140) in the NaCl 0.9% group (0.12, 0.30 and 0.26, respectively) and CYP group (0.10, 0.39 and 0.38, respectively) (Figs. 1c, 2c). The alterations of testicular tissues observed at D6 in mice treated with VCR seemed to persist over time, with a 2.1-fold higher global lesional score in VCR group (0.46 vs 0.22; *P* = 0.0017) than in the NaCl 0.9% group at D140 (Fig. 2d), resulting in the presence of vacuoles and pyknotic nuclei in seminiferous tubules (Fig. 2e). Vacuoles were also observed in testicular tissues of mice exposed to CYP (Fig. 2e) but the global lesional scores were not significantly different between CYP-treated mice and the NaCl 0.9% group at D66 and D140 (Fig. 2d). Moreover, less morphological alterations were detected between the age of D66 and D140 in CYP-treated mice (0.38 vs 0.17; *P* = 0.0022) (Fig. 2d).

### Disturbed progression of spermatogenesis after VCR exposure during the prepubertal period

**Figure 3:**
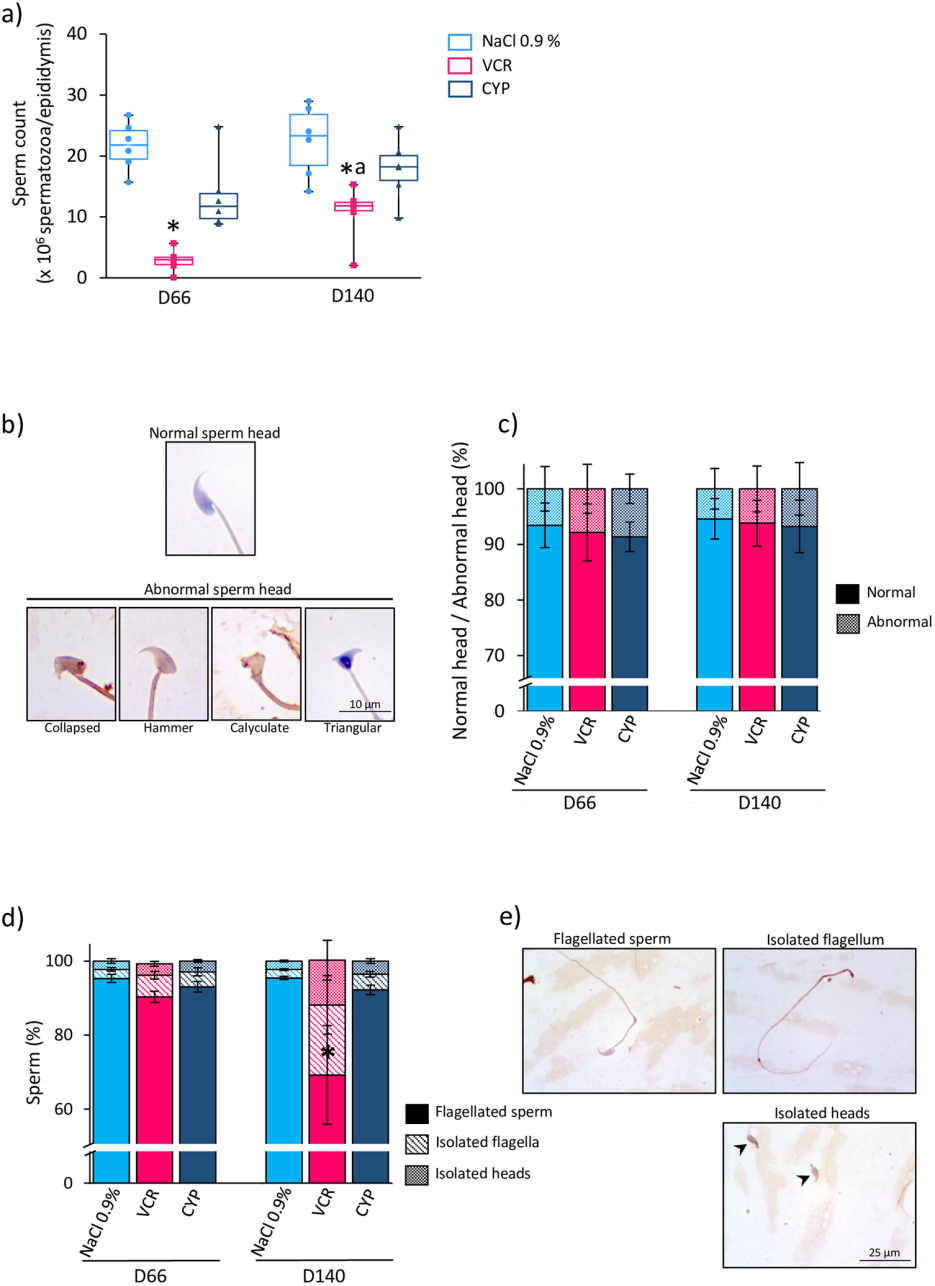
Sperm count and morphology in D66 and D140 mice treated by VCR or CYP during the prepubertal period. (**a**) Median sperm counts of D66 and D140 mice treated by NaCl 0.9%, VCR or CYP at 3 days of age are represented. (**b**) Epididymal sperm head morphology was classified into two groups: normal or abnormal (collapsed, hammer, calyculate or triangular). (**c**) Proportions of spermatozoa with normal or abnormal sperm head morphology in D66 and D140 mice exposed to chemotherapy (VCR or CYP) or unexposed (NaCl 0.9%). The percentages of flagellated spermatozoa, isolated flagella and isolated heads are represented for each condition (**d**). Representative photomicrographs of spermatozoa stained with haematoxylin are shown (**e**). Magnification: × 1000. Median is indicated by an horizontal line and points represent individual data with n = 6 mice for NaCl 0.9%, VCR and CYP groups for sperm count analyses. Data are presented as mean ± SEM with n = 6 mice (except for VCR at D66 where n = 5) for sperm morphology analyses. *P < 0.05 compared to NaCl 0.9% group at the same age. ^a^*P* < 0.05 compared to VCR group at D66.

The progression of spermatogenesis was assessed by determining the proportion of seminiferous tubules containing germ cells at the most advanced differentiation stage (Table 1). In all groups, the most advanced stage observed in tubules was elongated spermatids (Table 1) and spermatozoa were found in cauda epididymis, thereby showing the achievement of complete spermatogenesis (Fig. 3a). One VCR-treated male was considered azoospermic since no sperm was found in the epididymis at D66 (Fig. 3a) and therefore, could not be included in the analyses of sperm morphology and nuclear quality. This male was the only one to present Sertoli cell-only tubules at D66 (0.3 ± 0.3%). Tubules with no germ cells were also found in VCR-treated mice at D140, but no significant difference could be found relative to the NaCl 0.9% group, probably due to inter-individual variability in response to drugs (Table 1). At D66, a similar proportion of tubules at the most advanced stage was observed in all conditions, except in D66 VCR-treated mice where a significantly higher percentage of tubules containing pachytene spermatocytes (*P* = 0.0325) was found relative to the NaCl 0.9% group. In D140 VCR-treated mice, the progression of spermatogenesis was disturbed with a significantly raised percentage of tubules containing leptotene/zygotene spermatocytes (*P* = 0.0105), pachytene spermatocytes (*P* = 0.0025) and round spermatids (*P* < 0.0001) at the most advanced stage compared to the NaCl 0.9% group. Moreover, the proportion of tubules containing elongated spermatids was lower in VCR-treated males at D140 than in the NaCl 0.9% group (72.2 vs 91.1; *P* < 0.0001). The proportion of tubules in which round spermatids and elongating spermatids are the most advanced types of germ cells significantly increased between D66 and D140 in VCR-treated mice, whereas the proportion of tubules at the elongated spermatid stage significantly decreased (Table 1), thereby suggesting that the impact on spermatogenesis persisted after several cycles. In line with this, a decreased sperm count was observed at D66 (2.98 × 10^6^ vs 21.80 × 10^6^; *P* = 0.0013) and D140 (11.81 × 10^6^ vs 23.31 × 10^6^; *P* = 0.0199) in males exposed to VCR compared to the NaCl 0.9% group (Fig. 3a). A rise in sperm count was noticed in VCR-treated males between D66 and D140 (2.98 × 10^6^ vs 11.81 × 10^6^; *P* = 0.026). In contrast, sperm counts were not significantly different between CYP- and NaCl 0.9%-treated males (Fig. 3a).

**Table 1:** Percentage of seminiferous tubules at the most advanced differentiation stage of spermatogenesis in D66 and D140 mice exposed to chemotherapy (VCR or CYP) or unexposed (NaCl 0.9%) during the prepubertal period.

	% of seminiferous tubules at the most advanced stage					
	D66			D140		
	NaCl 0.9%	VCR	CYP	NaCl 0.9%	VCR	CYP
No germ cell (Sertoli cell-only)	0.0	0.3 ± 0.3	0.0	0.0	0.6 ± 0.4	0.0
Spermatogonia	0.0	0.0	0.0	0.0	0.6 ± 0.4	0.0
Leptotene/zygotene spermatocytes	0.0	0.0	0.0	0.0	1.4 ± 0.6*	0.0
Pachytene spermatocytes	0.6 ± 0.4	3.3 ± 1.0*	0.3 ± 0.3	0.3 ± 0.3	2.8 ± 0.7*	0.6 ± 0.4
Round spermatids	3.6 ± 1.0	7.5 ± 2.5	3.9 ± 1.1	3.3 ± 0.8	15.0 ± 2.0*^a^	4.7 ± 0.8
Elongating spermatids	3.9 ± 1.0	3.6 ± 0.5	4.7 ± 1.2	5.3 ± 0.8	7.5 ± 1.4 ^a^	7.2 ± 1.1
Elongated spermatids	92.2 ± 1.1	85.8 ± 3.1	91.1 ± 1.5	91.1 ± 1.1	72.2 ± 2.1*^a^	87.8 ± 1.5

Data are presented as mean ± SEM with n = 6 mice for NaCl 0.9%, VCR and CYP groups. *P < 0.05 compared to NaCl 0.9% group at the same age.

^a^*P* < 0.05 compared to VCR group at D66.

### Sperm morphological and nuclear abnormalities following CYP or VCR prepubertal exposure

Sperm head morphology was not affected by VCR and CYP exposure, with a similar proportion of spermatozoa with normal head compared to the NaCl 0.9% group at D66 and D140 (Fig. 3b,c). However, an increased percentage of isolated flagella was found in D140 VCR-treated mice (18.93 vs 2.27; *P* = 0.0154) in comparison with the NaCl 0.9% group (Fig. 3d,e).

**Figure 4:**
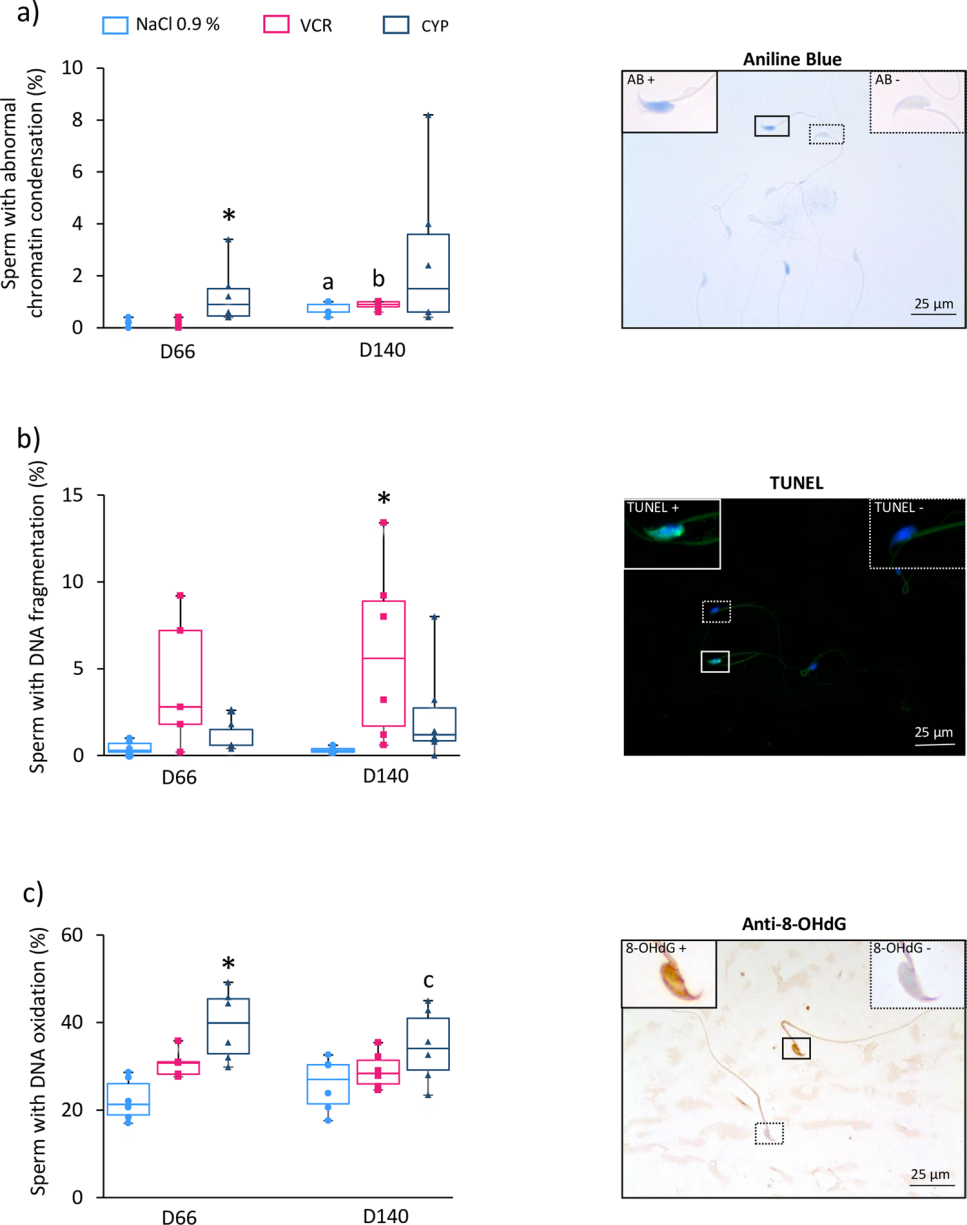
Impact of CYP or VCR prepubertal exposure on mouse sperm nuclear quality at D66 and D140. (**a**) Chromatin condensation in epididymal spermatozoa was assessed by aniline blue staining. Shown are representative images of spermatozoa with condensed (AB−, upper right corner) or abnormally condensed chromatin (AB+, upper left corner). Spermatozoa were counterstained with haematoxylin. The percentages of spermatozoa with abnormal chromatin condensation in D66 and D140 mice exposed to chemotherapy (VCR or CYP) or unexposed (NaCl 0.9%) are represented in box plots. (**b**) DNA fragmentation was investigated by TUNEL assay. Representative photomicrographs of spermatozoa with unfragmented DNA (TUNEL−, upper right corner) or fragmented DNA (TUNEL+ in green, upper left corner) are shown. Sperm nuclei were counterstained with DAPI (blue). Box plots show the proportions of spermatozoa with DNA fragmentation for NaCl 0.9%, VCR and CYP groups. (**c**) DNA oxidation was evaluated by 8-OHdG immunostaining. Representative images of spermatozoa with unoxidized DNA (8-OHdG−, upper right corner) and oxidized DNA (8-OHdG+, upper left corner) are shown. Spermatozoa were counterstained with haematoxylin. The percentages of 8-OHdG + spermatozoa are represented for each condition. Magnification: × 500 (Aniline blue staining and 8-OHdG immunostaining) or × 400 (TUNEL assay). Median is indicated by an horizontal line and points represent individual data with n = 6 mice for all conditions, except for VCR at D66 where n = 5. *P < 0.05 compared to NaCl 0.9% group at the same age. ^a^*P* < 0.05 compared to NaCl 0.9% group at D66. ^b^*P* < 0.05 compared to VCR group at D66. ^c^*P* < 0.05 compared to CYP group at D66.

After VCR and CYP exposure, an increased proportion of spermatozoa with nuclear abnormalities was observed. Indeed, aniline blue staining showed a higher percentage of spermatozoa with hypocondensed chromatin in D66 CYP-treated mice relative to the NaCl 0.9% group (0.90 vs 0.20; *P* = 0.0413) (Fig. 4a). In contrast, the percentage of spermatozoa with abnormal chromatin condensation was not different between males treated by VCR and NaCl 0.9% at D66 and D140. The proportion of spermatozoa with hypocondensed chromatin significantly increased between D66 and D140 in the VCR and NaCl 0.9% groups (Fig. 4a). TUNEL analyses also show a 18.6-fold increase in the proportion of spermatozoa containing fragmented DNA in D140 VCR-treated mice (5.6 vs 0.30; *P* = 0.0079) compared to the NaCl 0.9% group, but no significant difference was found for CYP-treated mice (Fig. 4b). Finally, more spermatozoa with DNA oxidation were found in CYP-treated mice at D66 compared to NaCl 0.9%-treated mice (39.90 vs 21.30; *P* = 0.0013) (Fig. 4c) whereas a similar proportion of spermatozoa with oxidized DNA was found in males treated by VCR and NaCl 0.9% (Fig. 4c).

### VCR or CYP treatment before puberty resulted in decreased fertility but had no impact on progeny development

**Figure 5:**
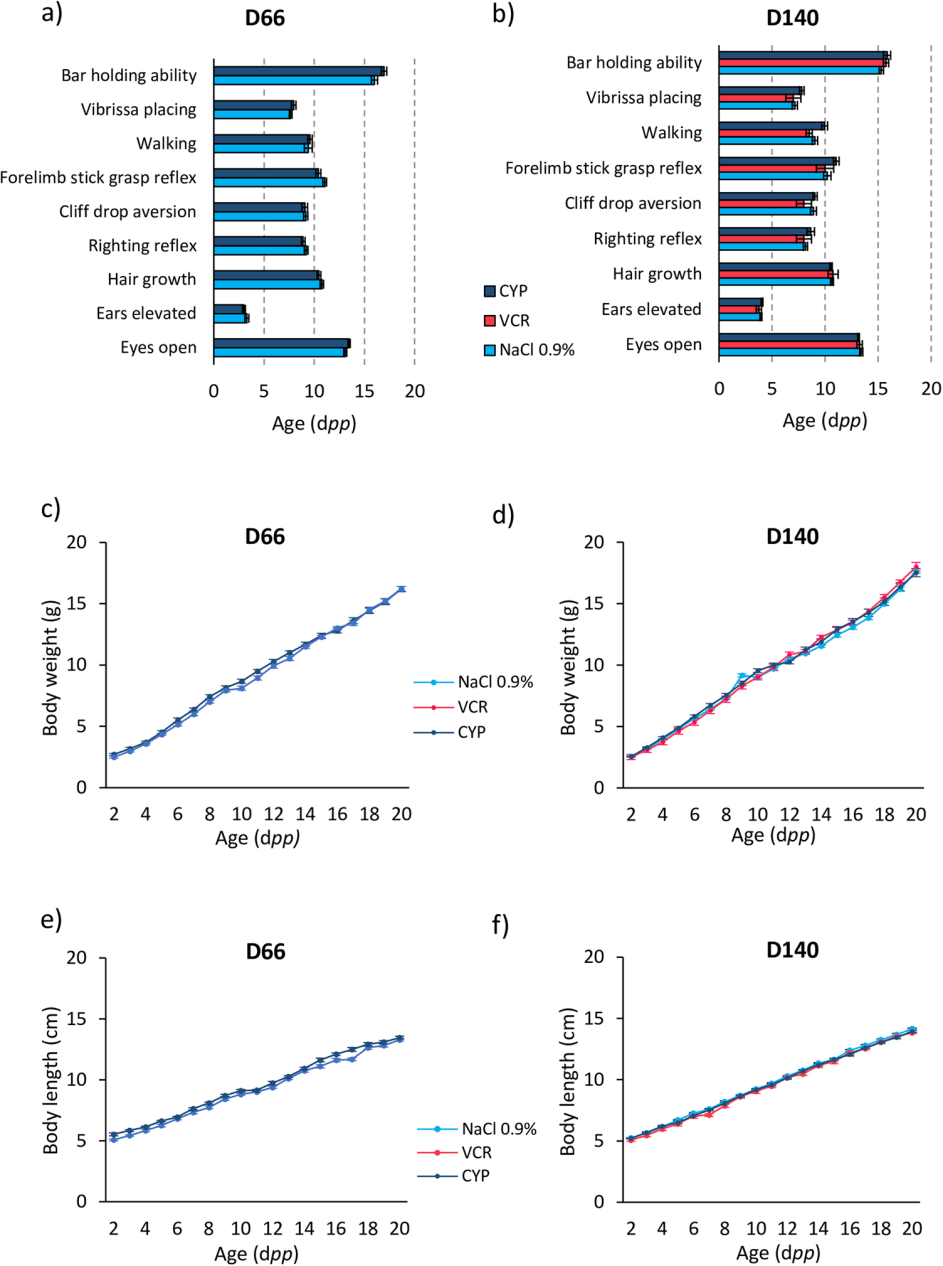
Impact of paternal exposure to chemotherapy (VCR or CYP) during the prepubertal period on the development of progeny. For each group, the pups obtained after mating D66 males (**a**) or D140 males (**b**) with untreated females were observed daily from 2 to 20 d*pp*. Their morphological (hair growth, ears elevation, eyes opening) and behavioural development (bar holding ability, vibrissa placing, walking, forelimb stick grasp reflex, cliff drop aversion, righting reflex) was assessed. The bar graphs show the mean age at which the pups are able to develop a mature and full response (level 3). The mean body weight of pups fathered by D66 males (**c**) or D140 males (**d**) was recorded during the pre-weaning period (from 2 to 20 d*pp*). The mean body length of pups fathered by D66 males (**e**) or D140 males (**f**) was measured from the age of 2 d*pp* to 20 d*pp*. Data are presented as mean ± SEM. For pups fathered by D66 males, n = 20 animals were observed in NaCl 0.9% group and n = 19 in CYP group. For pups fathered by D140 males, n = 20 animals were observed in all groups except for VCR group where n = 4.

**Figure 6:**
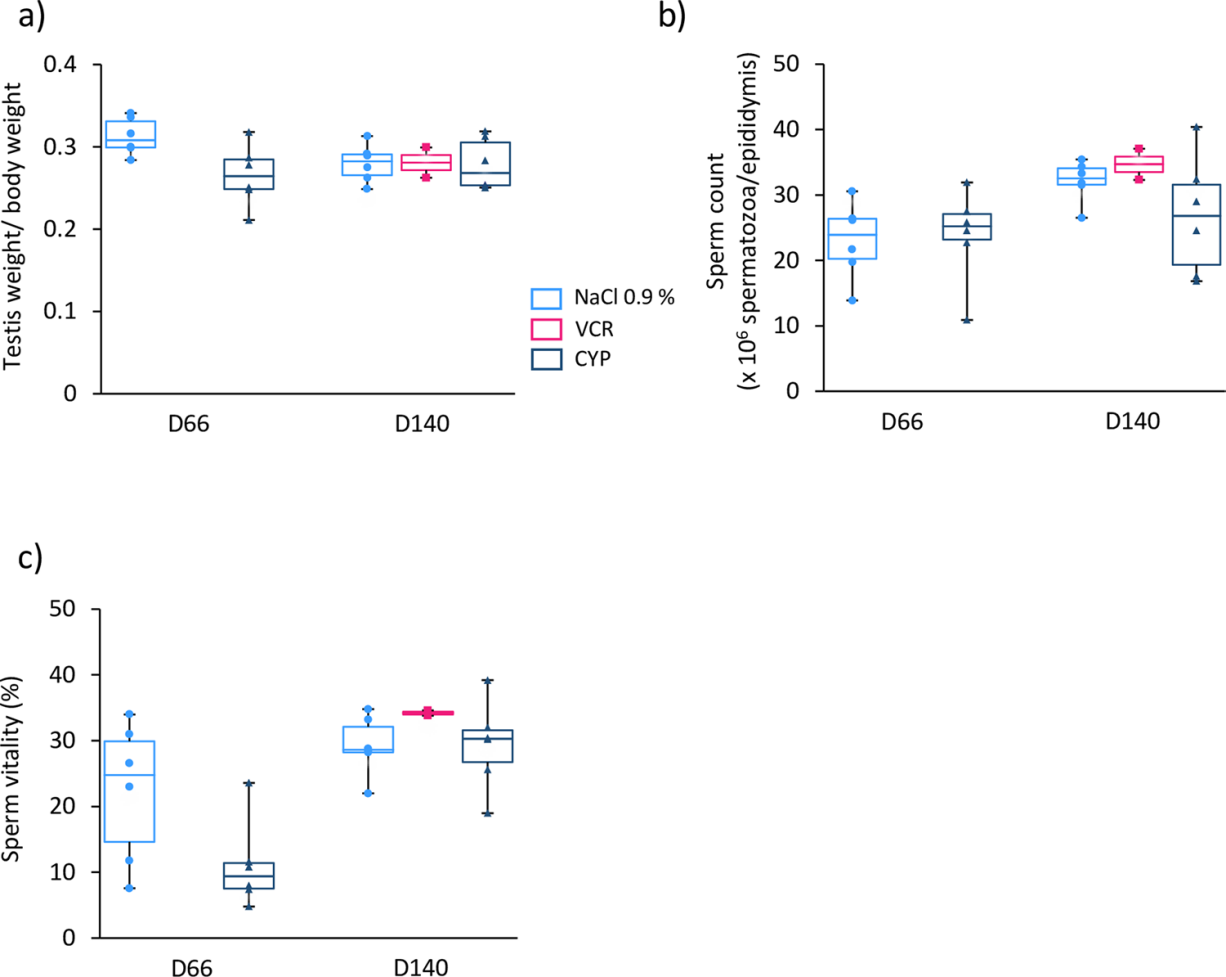
Impact of paternal exposure to VCR or CYP during the prepubertal period on the testes and sperm parameters of the male progeny. (**a**) Median ratio of testicular weight to body weight of mice fathered by D66 males and D140 males exposed to chemotherapy (VCR or CYP) or unexposed (NaCl 0.9%). (**b**) Median sperm counts were assessed at the age of sexual maturity in the offspring of D66 and D140 males. (**c**) Proportion of live sperm for each condition. Sperm vitality was evaluated by eosin-nigrosin staining at the time of collection. Median is indicated by an horizontal line and points represent individual data. For the male progeny fathered by D66 males, n = 6 animals were analysed in all groups. For the male progeny fathered by D140 males, n = 6 animals were analysed in all groups except for VCR group where n = 2.

Among the females mated with CYP-treated males at the age of D66 and D140, a significant reduction of pregnancy rate (number of pregnant females/total number of mated females × 100) was observed: 100% of the females became pregnant after mating with D66 and D140 NaCl 0.9%-treated mice versus 41.6% of pregnant females after mating with CYP-treated males at D66 (*P* = 0.002) and 50% at D140 (*P* = 0.005) (Table 2). No significant difference in post-implantation loss was found between the different groups at D66 and D140. Paternal CYP treatment had no impact on litter size, post-natal mortality and sex ratio. One litter was obtained with a D140 VCR-treated male (8.3% of pregnancy rate for the VCR group vs 100% for the NaCl 0.9% group, *P* < 0.0001). An important loss of post-implantation embryos was noticed after mating with VCR-treated males. The single litter fathered by the VCR-treated male only contained 7 pups, with a 2.5:1 male-to-female sex ratio (Table 2). No post-natal mortality was observed in this litter (Table 2). Moreover, external malformations were searched in all litters by investigating limb defects, body shape alterations or congenital anomalies (spina bifida, microcephaly and orofacial clefting) and no pups showed grossly visible anomalies. Pups were subjected to a set of behavioural tests and morphological screenings. Pups fathered by D66 and D140 males showed similar morphological (hair growth, ears elevation, eyes opening) and behavioural (bar holding ability, vibrissa placing, walking, forelimb stick grasp reflex, cliff drop aversion and righting reflex) developments in all groups (Fig. 5a,b). Paternal treatments did not impact the weight (Fig. 5c,d) and growth (Fig. 5e,f) of the offspring. At the age of sexual maturity, male descendants did not show any decrease in their testis weight (Fig. 6a) and any alteration in their sperm count (Fig. 6b) and sperm vitality (Fig. 6c).

**Table 2:** The effects of paternal VCR or CYP exposure before puberty on progeny outcome.

	D66			D140		
	NaCl 0.9%	VCR	CYP	NaCl 0.9%	VCR	CYP
Pregnacy rate (%)	100.0	0.0*	41.6*	100.0	8.3*	50.0*
Litter size (mean ± SEM)	14.1 ± 0.7	–	14.0 ± 0.6	13.6 ± 0.6	7.0 ± 0.0	14.2 ± 0.5
Post-implantation loss (%)	0.8	0.0	1.3	0.3	46.2	0.3
Post-natal mortality (%)	0.7	–	2.4	0.0	0.0	0.0
Sex ratio (m/f)	1.2	–	1.0	1.1	2.5	0.7

For each condition, six D66 or D140 male was mated with 12 virgin females. At the end of the gestation, pregnancy rate, litter size, post-natal mortality and sex ratio were determined. At the end of the weaning period, females were euthanized and post-implantation loss was assessed.

For pregnancy rate and post-implantation loss, data were obtained with n = 12 females for each condition. For litter size, post-natal mortality and sex ratio, data were collected according to the number of litters obtained for each condition, i.e. n = 12 litters for NaCl 0.9% groups at the age of D66 and D140; n = 5 litters for CYP group at D66; n = 6 litters for CYP group at D140 and n = 1 litter for VCR group at D140. No litter was obtained after mating of VCR-treated D66 males. *P < 0.05 compared to NaCl 0.9% group at the same age.

## Discussion

In the present study, the effects of VCR and CYP, two drugs commonly used during induction and consolidation chemotherapy at doses presumed with low gonadotoxicity, were investigated after prepubertal exposure in the mouse model. The doses of VCR and CYP used in the present study correspond to the highest doses that could be administered to 3 d*pp* pups while respecting the limit points. Higher doses of chemotherapy resulted in the death of prepubertal mice and lower doses were not clinically relevant.

Only 3 days after VCR or CYP treatment, major alterations of the seminiferous epithelium (presence of vacuoles, pyknotic nuclei) were detected. The global lesional score was respectively 3.3- and 1.7-fold higher in VCR- and CYP-exposed mice than in NaCl 0.9%-treated mice. Testicular weight represented at least 0.10% of body weight at D6 and 0.25% at adulthood in CYP- and NaCl 0.9%-treated mice. However, in male exposed to VCR, the ratio of testis weight to body weight was 0.09% at D6 and only 0.11% at adulthood, which may reflect a disruption in testicular development, probably occurring during puberty.

The morphological alterations of the testicular tissue observed at D6 persisted at the adult age (D140) and were associated with reduced epididymal sperm count in males exposed to VCR. An in vitro study previously indicated that VCR exposure leads to mitotic arrest and increased cell death in a rat spermatogonial cell line with SSC characteristics^22^. Here, the reduced sperm production in VCR-treated mice could therefore be a consequence of VCR toxicity on SSC. In addition, we observed Sertoli cell-only tubules in the testicular tissues of VCR-treated mice at D66 and D140, with one male showing azoospermia. These observations are in line with a possible SSC depletion in some tubules after prepubertal exposure to VCR. So far, only the impact of an adulthood exposure to VCR had been studied: in this case, a significant alteration of the germinal epithelium and decreased sperm count were observed 35 days after the administration of a single dose of VCR in mice^24,25^. Although VCR-treated mice showed a complete spermatogenesis at D140 in our study, the progression of spermatogenesis was disturbed: indeed, more tubules were at the leptotene/zygotene or pachytene spermatocyte stage and at the round spermatid stage whereas less tubules were at the elongated spermatid stage in comparison with NaCl 0.9%-treated mice. During prophase I of meiosis, major mechanisms such as homologous recombination of chromosomes and meiotic sex chromosome inactivation are critical to the pursuit of spermatogenesis. Exposure to VCR may lead to failures in these processes, which may activate the pachytene checkpoint and cause pachytene arrest in some seminiferous tubules^26^. Post-meiotic arrest before the elongation process could be the consequence of defects in chromatin packaging, cytoplasmic extrusion, flagellum and acrosome formation^27,28^.

In contrast, we found that prepubertal exposure to CYP did not affect testicular tissue integrity, progression of spermatogenesis and sperm production at adulthood (D66 and D140). The alterations of the germinal epithelium observed only 3 days after CYP treatment were no longer apparent at adulthood. So far, only one study had investigated the impact of prepubertal CYP in vivo exposure in rats: no histological damage and spermatogenesis disruption were observed in the testicular tissue 2 weeks after treatment (40, 200 and 280 mg/kg for 2 weeks)^29^. These results may indicate a rapid recovery of the seminiferous epithelium after prepubertal CYP exposure when administered at low gonadotoxic doses. The similar sperm count uncovered in CYP-treated mice relative to NaCl 0.9% group suggests the absence of major impact on the spermatogonial pool. However, the genotoxicity of alkylating agents, such as CYP, is well-known. A loss of germ cells including spermatogonial stem cells has been found after 24 h of in vitro exposure of 5 d*pp* mouse testis to a mid-concentration of CYP (0.2 µg/mL)^20^. Moreover, studies have shown a depletion of the spermatogonial pool in prepubertal patients who received alkylating agents at Cyclophosphamide Equivalent Doses (CED) of 5.5 and 7.0 g/m^2^ (presenting moderate risk for fertility) as well as others drugs, such as high doses of anthracycline^17,18,30^. Spermatogonial number but also sperm concentration are negatively correlated to CED^6,16^. Indeed, a proportion of azoospermic men reaching 80% has been found among childhood cancer survivors treated with high doses (> 19 g/m^2^) of alkylating agent versus 33% using non-sterilizing doses (< 19 g/m^2^)^31^. In addition, it has been estimated that 89% of childhood cancer survivors who received a CED < 4000 mg/m^2^ were normozoospermic^16^. No change in spermatogonial number has been observed in prepubertal boys who did not receive alkylating agent^18^. The present study reports no case of azoospermia in mice treated with CYP at a low gonadotoxic dose and one male displayed azoospermia in VCR group. The decreased sperm count uncovered in VCR-treated mice indicated a VCR toxicity on germ cells, as previously reported in other studies^24,32,33^. The rise in sperm counts observed between D66 and D140 in the VCR group suggests a possible sperm recovery. However, it must be considered that the presence of an azoospermic VCR-treated male at D66 drastically decreased the median sperm count and sperm recovery therefore remains questionable.

Very few studies have focused on sperm nuclear abnormalities in adult survivors of childhood cancer^34,35^. Indeed, sperm DNA damage have mainly been highlighted in rodent models^36,37^. Here, we report sperm morphological abnormalities and DNA damage in adult mice after VCR or CYP prepubertal exposure. In CYP-treated mice, an elevated proportion of spermatozoa with hypocondensed chromatin and oxidized DNA was found at D66. As an alkylating agent, CYP directly induces DNA adducts but also in an indirect manner via oxidative stress^38,39^. The oxidative stress induced by CYP at the time of exposure could lead to the formation of 8-hydroxy-2′-deoxyguanosine (8-OHdG) residues in the germline that could persist in spermatozoa and throughout spermatogenesis cycles^36,40^. Moreover, the accumulation of DNA damage alters sperm chromatin remodeling during spermiogenesis and affect chromatin structure^41,42^. The extreme compaction of chromatin is essential to protect paternal genome and plays a major role in the epigenetic reprogramming of paternal DNA after fertilization^43,44^. Defects in sperm chromatin condensation could therefore affect fertilization and embryo development. In our study, TUNEL assays showed a higher percentage of spermatozoa with DNA fragmentation in VCR-treated mice at D66, suggesting a long-term genotoxicity of VCR. A rise in DNA damage had previously been reported using COMET assay in mice exposed to VCR at adulthood^24^. Sperm morphological abnormalities were also observed in VCR-treated males, with a higher proportion of decapitated sperm. In human, sperm DNA damage and decapitated spermatozoa are frequently associated with male infertility^45–47^. Here, we show that prepubertal exposure to CYP or VCR at a low gonadotoxic dose impaired male fertility. Only 41.6% and 50% of females were pregnant after mating with CYP-treated D66 and D140 males, respectively. Moreover, a pregnancy rate of 8.3% was found in female mated with D140 VCR-treated male. These results contradict the current gonadotoxic risk classification in human. However, the relevance of our findings in humans should be interpreted with caution since the sensitivity of the prepubertal testis to chemotherapy is likely to be different between humans and rodents. In our study, 3 d*pp* mouse testes contain gonocytes and spermatogonia whereas human prepubertal testes exclusively contain spermatogonia^48^. Gonocytes and spermatogonia are the targets of chemotherapeutic drugs since they are mitotically active from 3 to 5 d*pp* in mouse testes^23^. Moreover, the regimen used might also explain the differences in testicular damage observed between the two species. Whilst human chemotherapy regimen is generally administered intravenously with cumulative doses for long periods of time, prepubertal mice only received one intraperitoneal injection of chemotherapy because of their small sizes. At the time of injection, intra-abdominally located testes may have direct contact with drugs, which may increase gonadotoxicity compared to intravenous injections.

Paternal CYP exposure did not affect litter size, post-implantation loss, post-natal mortality and sex ratio. Moreover, no significant difference in the morphological and behavioural development of the offspring has been evidenced and males fathered by CYP-treated mice had normal sperm parameters. In human, studies on the offspring of childhood cancer survivors remain limited but they have suggested that paternal treatment did not increase the risk of congenital abnormalities, genetic diseases and abnormal karyotypes in the offspring^49–51^. In our study, only one female became pregnant after mating with a D140 VCR-exposed male. Etoposide, like VCR, is considered to have a low gonadotoxic potential, although an exposure to this drug during the prepubertal period leads to a reduced number of offspring in the rat model^52^. Similarly, we found that the size of the litter fathered by the D140 VCR-treated mouse was reduced, probably because of 46.2% of embryo loss. A decreased number of total and live implantations has been reported after an adult exposure of spermatogonia to VCR^24^. Paternal DNA damage could be at the origin of arrests of embryo development and post-implantation losses^42,53^. In this litter, the sex ratio was in favour of males. No post-natal mortality, external malformation and alteration in the post-natal development was detected. However, since only one litter was obtained in this study, no conclusion on the effect of prepubertal VCR exposure on pregnancy outcome can be drawn.

In conclusion, persistent sperm DNA damage and decreased fertility were uncovered in adult mice following prepubertal CYP exposure at a low gonadotoxic dose. Short- and long-term alterations of the testicular tissue, disturbed progression of spermatogenesis associated with altered sperm morphology and nuclear quality were evidenced after prepubertal VCR treatment. Contrary to the current gonadotoxic risk classification in human, our data using a mouse model show a decreased fertility of more than 50% and 90% in males treated with a ‘low gonadotoxic’ dose of CYP and VCR, respectively. The administration of these drugs during induction and consolidation chemotherapy could therefore have long-term adverse effects on fertility in paediatric cancer survivors who have not benefited from a fertility preservation procedure. Moreover, the unrepaired DNA damage following prepubertal chemotherapy exposure might persist and could decrease the chances of fertility restoration for patients who have benefited from the experimental procedure of testicular tissue cryopreservation.

While further research on the impact of chemotherapy on the prepubertal testis is urgently required, human samples remain scarce. Data on animal models could guideline future studies in human and be valuable to investigate the mechanism of action of chemotherapy agents, either administered alone or in combination. These data will help to better inform the paediatric cancer patients about the risks of chemotherapy for their future fertility and to propose fertility preservation options.

## Methods

### Animals, treatments and samples collection

All the animal procedures were approved by the Institutional Animal Care and Use Committee of Rouen Normandy University (under the protocol number APAFiS #18208) and carried out in accordance with French Ministry of Higher Education, Research and Innovation regulation. CD-1 mice (Charles River Laboratories, L’Arbresle, France) were housed on a 12 h light:12 h dark cycle, with food and water ad libitum. In order to respect the 3Rs principle, we have reduced our groups to n = 6 mice per group.

Animals were divided randomly into 4 different groups: Untreated, NaCl 0.9%, VCR and CYP groups (Fig. S2a). Mice in the untreated group did not receive any injection. Prepubertal 3-day-old mice either received saline solution (NaCl 0.9% group), cyclophosphamide (CYP group) or vincristine (VCR group). The treatments were administered intraperitoneally in a single dose of 200 µg/kg for vincristine (ONCOVIN, Teva Santé, Paris, France) or 90 mg/kg for cyclophosphamide (ENDOXAN, Baxter, Guyancourt, France). The drugs used in our study are the same as those employed in cancer treatment in humans, as well as the composition of the excipients. The doses chosen are similar to the low concentrations used in induction and consolidation chemotherapy in the clinics and are equivalent to 6 mg/m^2^ for VCR and 270 mg/m^2^ for CYP. They have been adapted to the murine model with a standard factor to interspecies doses conversion, as recommended by the Food and Drug Administration.

To analyse testicular tissue integrity and the progression of spermatogenesis, testes from 6 d*pp* (D6), 66 d*pp* (D66) and 140 d*pp* (D140) mice were obtained after euthanasia (by decapitation for D6 mice and by CO_2_ asphyxiation for D66 and D140 mice). The tunica albuginea was removed with two needles under a binocular magnifier (S8AP0, Leica Microsystems GmbH, Wetzlar, Germany) before the fixation of mouse testes in Bouin’s solution (2 h for D6 and 7 h for D66 and D140). Testes were dehydrated in graded series of ethanol and xylene washes and embedded in paraffin. Tissue sections (3 µm thick) were cut using a microtome (JungRM 2035; Leica Microsystems GmbH) and mounted on Polysine slides (Thermo Fischer Scientific, Saint-Aubin, France). The left cauda epididymis of sexually mature D66 and D140 mice was used for sperm collection. Spermatozoa were extracted from the cauda epididymis by gentle pressure with needles in M16 medium (Sigma-Aldrich, Saint-Quentin Fallavier, France). Twenty microliters of the suspensions were used to determine sperm counts using a Thoma cell counting chamber under a light microscope (DM4000B, Leica Microsystems GmbH). Sperm count was performed by counting all spermatozoa in 12 medium squares of 0.04 mm^2^ area in the hemocytometer counting chamber. The rest of the suspensions was centrifuged for 10 min at 300×*g*. Pellets containing spermatozoa were recovered and either fixed with methanol (1 h, 4 °C) for Terminal deoxynucleotidyl transferase mediated dUTP nick end labelling (TUNEL) assays or with 4% (w/v) paraformaldehyde (30 min, 4 °C) for the immunocytochemical detection of 8-hydroxy-2′-deoxyguanosine (8-OHdG). Spermatozoa were then spread onto glass slides. For aniline blue staining, spermatozoa were resuspended in PBS solution before spreading onto slides and fixed with 3% (v/v) glutaraldehyde (Sigma-Aldrich) (30 min, RT).

### Histological analyses

Testicular tissue sections were deparaffinized in xylene and rehydrated with decreasing concentrations of ethanol. Hemalun eosin saffron (HES) staining was performed to visualize seminiferous tubule architecture, as previously described^54^ and periodic acid Schiff reaction (RAL diagnostic, Martillac, France) was used to detect the pink-labelled acrosome of spermatids and visualize the progression of spermatogenesis. Tissue structural integrity was assessed in each HES-stained testicular tissue section by calculating a global lesional score on a scale from 0 to 10 (0–5 for epithelial integrity and 0–5 for nuclear alteration, with 0 representing the complete absence of alteration and 5 representing the most important damage)^54^.

Analyses were conducted with a light microscope (DM4000B, Leica Microsystems GmbH) equipped with a Leica Application Suite software. For each animal, the global lesional score was obtained from 60 cross-sectioned tubules in 2 sections separated by an interval of at least 35 µm.

### Sperm analyses

Spermatozoa fixed with 4% (w/v) paraformaldehyde were stained with haematoxylin (Dako, Les Ulis, France). A modified version of the sperm head morphology classification developed by Burruel et al*.*^55^ was used to screen sperm head abnormalities. Spermatozoa were assigned to two categories: normal head and abnormal head. Abnormal heads included collapsed, triangular, calyculate and hammer heads. The percentages of isolated flagella, isolated heads and flagellated spermatozoa were also determined.

Sperm chromatin condensation was assessed following aniline blue staining. After a 15-min staining with 5% (w/v) aniline blue (Sigma-Aldrich) at pH 3.5, spermatozoa were counterstained with haematoxylin for 15 s. Slides were then mounted with Eukitt (CML, Nemours, France).

Sperm DNA oxidation was assessed by the immunodetection of 8-OHdG. After fixation with 4% (w/v) paraformaldehyde, sperm DNA was decondensed with 1 M NaOH for 5 min. Spermatozoa were incubated in 5% (w/v) bovine serum albumin (BSA, Sigma-Aldrich) in PBS for 30 min and in HP Block (Dako) for 5 min at RT to block non-specific binding sites and endogenous peroxidases, respectively. Incubation with mouse monoclonal anti-8-OHdG IgGs (2 μg/mL in PBS + 5% (w/v) BSA, clone 15A3, Novus Biologicals, Littleton, CO, USA) was conducted overnight at 4 °C. Then, spermatozoa were incubated with biotinylated goat polyclonal anti-mouse IgGs (TP-060-BN, Thermo Fisher Scientific) for 5 min at RT and with horseradish peroxidase-conjugated streptavidin (TS-060-QPH, Thermo Fisher Scientific) for 5 min. Immunological labelling was revealed after incubation with 3,3′-diaminobenzidine (1:50, Thermo Fischer Scientific) for 1 min. Slides were counterstained with haematoxylin for 15 s and mounted. Negative controls were performed with pre-immune mouse IgGs (SC-2025, SantaCruz Biotechnology, Heidelberg, Germany). Positive controls were performed by incubating spermatozoa with 30% (w/v) H_2_O_2_ for 30 min at RT prior to fixation and 8-OHdG immunostaining.

TUNEL assays were carried out to detect spermatozoa with fragmented DNA. After a 2-min permeabilization in acetone, TUNEL assays were performed using the In Situ Cell Death Detection kit POD (Roche, Mannheim, Germany) for 1 h at 37 °C, following manufacturer’s instructions. Slides were mounted in Vectashield with DAPI (4′,6-diamidino-2-phenylindole). Positive controls were performed by incubating spermatozoa with DNase I for 15 min at 37 °C before TUNEL assays and negative controls were carried out by omitting the enzyme solution.

For sperm morphology analyses, aniline blue staining and 8-OHdG immunostaining, slides were examined under a DM4000B light microscope (Leica Microsystems GmbH) at a 1000 × magnification. Sperm DNA fragmentation was analysed with an Axioskop fluorescence microscope equipped with an AxioCam 503 camera (Carl Zeiss SAS, Marly-le-Roi, France) at a 400 × magnification. For each analysis, 500 spermatozoa were counted per animal with n = 6 mice for all groups except for VCR at D66 where n = 5.

### Analysis of pregnancy outcome and behavioural testing of offspring

Each male was mated with 2 females; i.e. six males at the age of D66 or D140 were mated with 12 virgin females for each group (Fig. S2b). The mated females were then individually housed. On the day of birth, the number of pups per litter (litter size), sex of pups (sex ratio) and pregnancy rate (number of pregnant females/total number of mated females × 100) were determined in all the litters obtained (58 litters in total). Pups were inspected for any malformation and the number of dead mice was recorded. Five litters per condition were saved except for VCR group where only one litter was obtained. To ensure equality in nutrition and growth rates, the litter size was reduced to 2 females and 2 males from the second day after birth. Pups were weighed daily (from day 2 to 20) and were inspected to evaluate their behavioural and morphological development until the end of the weaning period. This evaluation was carried out according to Van der Meer et al.^56^, i.e. each behaviour test was associated with four levels of response: 0 (no response), 1 (low response), 2 (a clear but not yet mature response) or 3 (a mature and full response). The development of locomotion (walking), labyrinth response (righting reflex), somato-sensory response (cliff drop aversion), freeing response (forelimb stick grasp reflex) and muscular strength (bar holding ability) were assessed. Morphological development was also monitored through hair growth, ears elevation and eyes opening. At the end of the weaning period, mothers were euthanised by CO_2_ asphyxiation and uteri were removed. Incubation of uteri in 2% (v/v) ammonium sulfide solution (Sigma-Aldrich) for 2 min revealed implantation sites. Post-implantation loss (number of implantation sites − number of live fetuses/total number of implantations) was calculated for 96 females in total (n = 12 for each group).

### Analysis of the reproductive organs of the male progeny

Among the pups of each condition, six sexually mature males were randomly selected and euthanized (Fig. S2b). Only two males fathered by the D140 VCR-treated mice have been investigated. Testes were weighed and the left cauda epididymis were removed for sperm counts, as described above. Sperm vitality was assessed following eosin-nigrosin staining. Sperm suspensions were mixed with 1 volume of 1% (w/v) eosin for 30 s and with 2 volumes of 10% (w/v) nigrosin for 40 s. Suspensions were smeared onto slides in order to determine the percentages of live and dead spermatozoa. For sperm vitality analyses, 500 spermatozoa were examined per animal under a DM4000B light microscope (Leica Microsystems GmbH) at a 1000 × magnification.

### Statistical analyses

Statistical analyses were carried out with the GraphPad Prism v6.0 software (GraphPad Software Inc., La Jolla, CA, USA). Non-parametric Kruskall–Wallis tests followed by Dunn’s post hoc tests were performed to assess statistical differences between untreated, NaCl 0.9%, VCR and CYP groups. The non-parametric Mann–Whitney test was used to determine statistical significances between D66 and D140 within a group. The Chi^2^ test was performed to assess statistical differences for pregnancy rate between all the groups. Results are expressed as median (min–max) or mean ± SEM. *P* < 0.05 was considered statistically significant.

## Supplementary information

Supplementary Figures.
