# Supplementary material for: Paradoxical risk of reduced fertility after exposure of prepubertal mice to vincristine or cyclophosphamide at low gonadotoxic doses in humans
Source: Sci Rep. 2020 Oct 20;10:17859. doi: 10.1038/s41598-020-74862-8 (PMC7576200; doi:10.1038/s41598-020-74862-8)
Supplement: Supplementary file 1 — Supplementary Figures. [file 41598_2020_74862_MOESM1_ESM.pdf]

Running title: Chemotherapy-induced prepubertal gonadotoxicity

Title: Paradoxical risk of reduced fertility after exposure of prepubertal mice to vincristine or cyclophosphamide at low gonadotoxic doses in humans

Marion Delessard, Justine Saulnier, Ludovic Dumont, Aurélie Rives-Feraille, Nathalie Rives, Christine Rondanino

Normandie Univ, UNIROUEN, EA 4308 "Gametogenesis and Gamete Quality", Rouen University Hospital, Department of Reproductive Biology–CECOS, F 76000 Rouen, France

Corresponding author: Dr Christine Rondanino, Ph.D.; email: christine.rondanino@univ-rouen.fr; phone: +33 2 35 14 82 94.

Key words: chemotherapy, prepubertal exposure, mouse fertility, vincristine, cyclophosphamide

Supplementary figure 1. Statistical comparisons between untreated and NaCl 0.9% groups

Mice in NaCl 0.9% group received an intraperitoneal injection of saline solution at D3 and mice in untreated group did not receive any injection. The data obtained on prepubertal testes (**a**), adult testes, gametes, fertility and progeny (**b**) in both groups and the statistical differences between the two groups are presented. The mean body weight of pups fathered by D66 males or D140 males (**c**) as well as their mean body length (**d**) are also reported. Non-parametric Kruskall-Wallis tests followed by Dunn's *post hoc* tests were performed to assess statistical differences between untreated and NaCl 0.9% groups for all the data, except for pregnancy rate where Chi^2 test was performed. For all the data obtained in this study, the NaCl 0.9% group showed no significant difference with the untreated group.

a)

	D6		
	Untreated	NaCl 0.9%	P-value
**Prepubertal testes**<br>Data are represented as median (min – max)			
Body weight (g)	5.03 (4.79 – 5.20)	4.82 (4.69 – 6.34)	> 0.9999
Testis weight (mg)	5.28 (4.20 – 7.40)	6.15 (4.45 – 7.75)	> 0.9999
Testis weight / Body weight	0.11 (0.08 – 0.11)	0.12 (0.09 – 0.12)	> 0.9999
Global lesional score	0.18 (0.10 – 0.52)	0.19 (0.03 – 0.23)	> 0.9999

b)

	D66			D140		
	Untreated	NaCl 0.9%	P-value	Untreated	NaCl 0.9%	P-value
**Adult testes**<br>Data are represented as median (min – max)						
Body weight (g)	38.07 (34.95 – 52.18)	47.55 (25.30 – 53.73)	> 0.9999	44.49 (42.65 – 54.26)	52.28 (46.72 – 73.53)	0.4954
Testis weight (mg)	126.18 (115.50 – 144.35)	126.95 (99.30 – 173.85)	> 0.9999	129.85 (115.75 – 140.90)	134.25 (128.95 – 147.45)	> 0.9999
Testis weight / Body weight	0.33 (0.23 – 0.44)	0.30 (0.23 – 0.44)	> 0.9999	0.27 (0.25 – 0.33)	0.26 (0.18 – 0.30)	> 0.9999
Global lesional score	0.33 (0.12 – 0.76)	0.35 (0.13 – 0.53)	> 0.9999	0.15 (0.06 – 0.36)	0.22 (0.16 – 0.29)	0.4576
**Percentage of seminiferous tubules at the most advanced stage**<br>Data are represented as mean ± SEM						
No germ cell (Sertoli cell-only)	0.00 ± 0.00	0.00 ± 0.00	> 0.9999	0.00 ± 0.00	0.00 ± 0.00	> 0.9999
Spermatogonia	0.00 ± 0.00	0.00 ± 0.00	> 0.9999	0.00 ± 0.00	0.00 ± 0.00	> 0.9999
Leptotene/zygotene spermatocytes	0.00 ± 0.00	0.00 ± 0.00	> 0.9999	0.00 ± 0.00	0.00 ± 0.00	> 0.9999
Pachytene spermatocytes	0.83 ± 0.44	0.56 ± 0.37	> 0.9999	0.28 ± 0.28	0.28 ± 0.28	> 0.9999
Round spermatids	5.28 ± 0.64	3.61 ± 0.96	0.6418	5.83 ± 0.93	3.33 ± 0.82	0.3226
Elongating spermatids	6.39 ± 1.12	3.89 ± 0.99	0.0754	5.28 ± 1.26	5.28 ± 0.76	> 0.9999
Elongated spermatids	87.50 ± 1.31	92.22 ± 1.11	0.0628	88.61 ± 0.87	91.11 ±1.11	0.6822
**Sperm count**<br>Data are represented as median (min – max)						
Sperm count (10^6 spermatozoa / epididymis)	20.09 (14.81 – 38.13)	21.80 (15.69 – 26.71)	> 0.9999	24.31 (22.67 – 35.83)	23.31 (14.17 – 29.00)	> 0.9999
**Sperm morphology**<br>Data are represented as mean ± SEM						
Normal sperm head (%)	94.53 ± 2.78	93.43 ± 4.00	0.6729	94.63 ± 3.99	94.60 ± 3.64	0.6729
Abnormal sperm head (%)	5.47 ± 2.78	6.57 ± 4.00	> 0.9999	5.37 ± 3.99	5.40 ± 3.64	> 0.9999
Flagellated sperm (%)	96.07 ± 0.67	95.30 ± 1.09	0.3108	96.77 ± 0.65	95.43 ± 0.41	0.7556
Isolated flagella (%)	2.00 ± 0.35	2.43 ± 0.49	> 0.9999	1.50 ± 0.41	2.27 ± 0.23	> 0.9999
Isolated heads (%)	1.93 ± 0.42	2.27 ± 0.63	> 0.9999	1.73 ± 0.33	2.30 ± 0.22	> 0.9999
**Sperm nuclear quality**<br>Data are represented as median (min – max)						
Sperm with abnormal chromatin condensation (%)	0.00 (0.00 – 0.80)	0.20 (0.00 – 0.40)	> 0.9999	0.40 (0.00 – 0.60)	0.60 (0.40 – 1.00)	> 0.9999

Sperm with DNA fragmentation (%)	0.50 (0.00 – 1.00)	0.30 (0.00 – 1.00)	> 0.9999	0.30 (0.20 – 1.20)	0.30 (0.20 – 0.60)	> 0.9999
Sperm with DNA oxidation (%)	29.20 (20.55 – 41.20)	21.30 (17.00 – 28.60)	0.2905	25.5 (15.80 – 37.20)	27.00 (17.60 – 32.60)	> 0.9999
**Progeny outcome**						
Pregnancy rate (%)	100	100	> 0.9999	83.33	100.00	0.1396
Litter size (mean ± SEM)	14.60 ± 0.75	14.1 ± 0.74	> 0.9999	15.10 ± 0.69	13.58 ± 0.57	0.2146
Post-implantation loss (%)	0.88	0.78	0.9444	0.27	0.30	> 0.9999
Post-natal mortality (%)	0	0.71	0.8573	0.00	0.00	> 0.9999
Sex ratio (m/f)	1.00	1.19	0.4592	1.31	1.14	0.4729
**Development of the progeny**<br>Data are represented as mean ± SEM						
Bar holding ability (d*pp*)	16.25 ± 0.30	16.00 ± 0.30	0.9709	15.80 ± 0.29	15.30 ± 0.21	0.7073
Vibrissa placing (d*pp*)	8.15 ± 0.21	7.65 ± 0.13	0.1856	7.30 ± 0.22	7.15 ± 0.25	> 0.9999
Walking (d*pp*)	9.85 ± 0.32	9.40 ± 0.39	> 0.9999	9.1 ± 0.26	9.05 ± 0.26	> 0.9999
Forelimb stick grasp reflex (d*pp*)	11.15 ± 0.19	11.05 ± 0.18	> 0.9999	10.95 ± 0.24	10.2 ± 0.35	0.0858
Cliff drop aversion (d*pp*)	9.75 ± 0.19	9.15 ± 0.21	0.1229	9.15 ± 0.27	8.90 ± 0.27	> 0.9999
Righting reflex (d*pp*)	8.90 ± 0.10	9.20 ± 0.18	0.4722	8.8 ± 0.09	8.15 ± 0.19	0.1228
Hair growth (d*pp*)	10.85 ± 0.15	10.75 ± 0.18	> 0.9999	11.05 ± 0.15	10.65 ± 0.13	0.1895
Ears elevated (d*pp*)	3.45 ± 0.11	3.30 ± 0.19	> 0.9999	3.75 ± 0.10	3.95 ± 0.09	0.4098
Eyes open (d*pp*)	13.30 ± 0.11	13.1 ± 0.15	0.3718	13.30 ± 0.11	13.4 ± 0.15	> 0.9999
**Testes and sperm parameters of the male progeny**<br>Data are represented as median (min – max)						
Testis weight / Body weight	0.29 (0.24 – 0.40)	0.31 (0.28 – 0.34)	0.7159	0.28 (0.26 – 0.32)	0.28 (0.25 – 0.31)	> 0.9999
Sperm count (10^6 spermatozoa / epididymis)	27.94 (18.33 – 38.00)	23.92 (13.92 – 30.56)	0.4072	24.69 (19.06 – 40.83)	32.56 (26.50 – 35.44)	0.5156
Sperm vitality (%)	17.90 (4.60 – 22.40)	24.80 (7.60 – 34.00)	0.5123	27.00 (8.20 – 30.20)	28.60 (22.00 – 34.80)	0.4916

c)

	Age (d*pp*)	**2**	**3**	**4**	**5**	**6**	**7**	**8**	**9**	**10**	**11**	**12**	**13**	**14**	**15**	**16**	**17**	**18**	**19**	**20**
**D66**	Untreated (g)	2.60 ± 0.06	3.11 ± 0.07	3.84 ± 0.09	4.52 ± 0.06	5.37 ± 0.11	6.31 ± 0.15	7.11 ± 0.13	7.75 ± 0.17	8.47 ± 0.18	9.33 ± 0.19	10.18 ± 0.15	10.96 ± 0.16	11.82 ± 0.11	12.31 ± 0.11	12.95 ± 0.11	13.83 ± 0.11	14.62 ± 0.12	15.57 ± 0.13	16.54 ± 0.13
	NaCl 0.9% (g)	2.49 ± 0.08	2.98 ± 0.07	3.58 ± 0.09	4.35 ± 0.07	5.15 ± 0.10	5.99 ± 0.13	7.01 ± 0.11	7.93 ± 0.12	8.11 ± 0.17	8.95 ± 0.17	9.93 ± 0.19	10.53 ± 0.17	11.51 ± 0.15	12.29 ± 0.14	13.01 ± 0.13	13.43 ± 0.22	14.51 ± 0.23	15.23 ± 0.21	16.23 ± 0.18
	P-value	0.5899	0.4687	0.1057	0.2855	0.4185	0.205	>0.9999	0.8937	0.4475	0.4507	0,6839	0.1373	0.2840	>0.9999	>0.9999	0.1599	>0.9999	0.2107	0.2428
**D140**	Untreated (g)	2.56 ± 0.05	3.34 ± 0.07	4.00 ± 0.08	4.88 ± 0.10	5.76 ± 0.11	6.64 ± 0.10	7.63 ± 0.12	8.47 ± 0.11	9.46 ± 0.12	9.91 ± 0.10	10.47 ± 0.12	11.35 ± 0.11	11.87 ± 0.13	12.53 ± 0.12	13.14 ± 0.12	14.36 ± 0.12	15.50 ± 0.16	16.48 ± 0.17	17.67 ± 0.21
	NaCl 0.9% (g)	2.56 ± 0.05	3.20 ± 0.05	3.97 ± 0.06	4.81 ± 0.06	5.66 ± 0.06	6.41 ± 0.07	7.28 ± 0.07	9.17 ± 0.13	9.02 ± 0.08	9.70 ± 0.07	10.50 ± 0.12	10.95 ± 0.08	11.55 ± 0.13	12.43 ± 0.16	13.08 ± 0.16	13.83 ± 0.13	14.98 ± 0.13	16.19 ± 0.19	17.66 ± 0.19
	P-value	>0.9999	0.1556	>0.9999	0.9555	0.8209	0.2107	0.083	0.1295	0.2962	0.3268	>0.9999	0.0809	0.3021	>0.9999	>0.9999	0.0777	0.1756	>0.9999	>0.9999

d)

	Age (d*pp*)	**2**	**3**	**4**	**5**	**6**	**7**	**8**	**9**	**10**	**11**	**12**	**13**	**14**	**15**	**16**	**17**	**18**	**19**	**20**
**D66**	Untreated (cm)	5.21 ± 0.07	5.71 ± 0.06	5.96 ± 0.10	6.47 ± 0.06	6.88 ± 0.10	7.38 ± 0.12	7.85 ± 0.09	8.64 ± 0.12	8.88 ± 0.12	9.06 ± 0.09	9.43 ± 0.13	10.19 ± 0.08	10.73 ± 0.07	11.44 ± 0.09	11.82 ± 0.08	12.20 ± 0.07	12.58 ± 0.06	12.90 ± 0.06	13.47 ± 0.08
	NaCl 0.9% (cm)	5.09 ± 0.07	5.43 ± 0.06	5.85 ± 0.09	6.26 ± 0.11	6.82 ± 0.09	7.35 ± 0.12	7.75 ± 0.10	8.44 ± 0.10	8.82 ± 0.09	9.03 ± 0.08	9.39 ± 0.09	10.10 ± 0.08	10.76 ± 0.12	11.12 ± 0.13	11.64 ± 0.14	11.70 ± 0.12	12.64 ± 0.12	12.82 ± 0.12	13.29 ± 0.11
	P-value	0.3607	>0.9999	0.7185	>0.9999	>0.9999	>0.9999	>0.9999	0.4220	>0.9999	>0.9999	>0.9999	>0.9999	>0.9999	0.3571	>0.9999	0.4809	>0.9999	0.5553	0.1874
**D140**	Untreated (cm)	5.11 ± 0.03	5.68 ± 0.05	6.17 ± 0.05	6.70 ± 0.07	7.23 ± 0.07	7.47 ± 0.14	8.27 ± 0.06	8.94 ± 0.10	9.41 ± 0.09	9.71 ± 0.08	10.35 ± 0.08	10.77 ± 0.08	11.37 ± 0.08	11.77 ± 0.08	12.35 ± 0.07	12.84 ± 0.07	13.35 ± 0.08	13.59 ± 0.07	14.08 ± 0.09
	NaCl 0.9% (cm)	5.23 ± 0.04	5.65 ± 0.04	6.14 ± 0.04	6.70 ± 0.05	7.25 ± 0.07	7.59 ± 0.06	8.20 ± 0.07	8.70 ± 0.07	9.23 ± 0.05	9.68 ± 0.06	10.29 ± 0.05	10.75 ± 0.07	11.33 ± 0.06	11.65 ± 0.09	12.38 ± 0.08	12.78 ± 0.08	13.25 ± 0.07	13.68 ± 0.09	14.15 ± 0.07
	P-value	0.0914	>0.9999	>0.9999	>0.9999	>0.9999	>0.9999	0.8145	0.2433	0.6122	>0.9999	>0.9999	>0.9999	>0.9999	0.5881	>0.9999	>0.9999	0.8998	>0.9999	>0.9999

Supplementary figure 2. Flow chart of the study design

The effects of *in vivo* exposure of prepubertal mice to VCR or CYP administered individually at 3-day *postpartum* (D3) were analysed in the short-term (D6) and the long-term (D66 and D140) (**a**). The integrity of testicular tissues, sperm production and sperm nuclear quality were assessed for each condition: untreated, NaCl 0.9%, VCR and CYP groups.

Fertility, pregnancy outcome and behavioural development of the offspring were examined after mating of one D66 or D140 male with 2 untreated females for each condition (**b**). Post-implantation loss and pregnancy rate were determined for all the mated females. On the day of birth, litter size, post-natal mortality and sex ratio were determined in all the litters obtained. To ensure equality in nutrition and growth rates, the number of litters was reduced to 5 per group with 2 females and 2 males in each litter. Pups were inspected to evaluate their behavioural and morphological development until the end of the weaning period. Among the 5 litters of each condition, six sexually mature males were randomly selected to assess reproductive organ and sperm parameters.

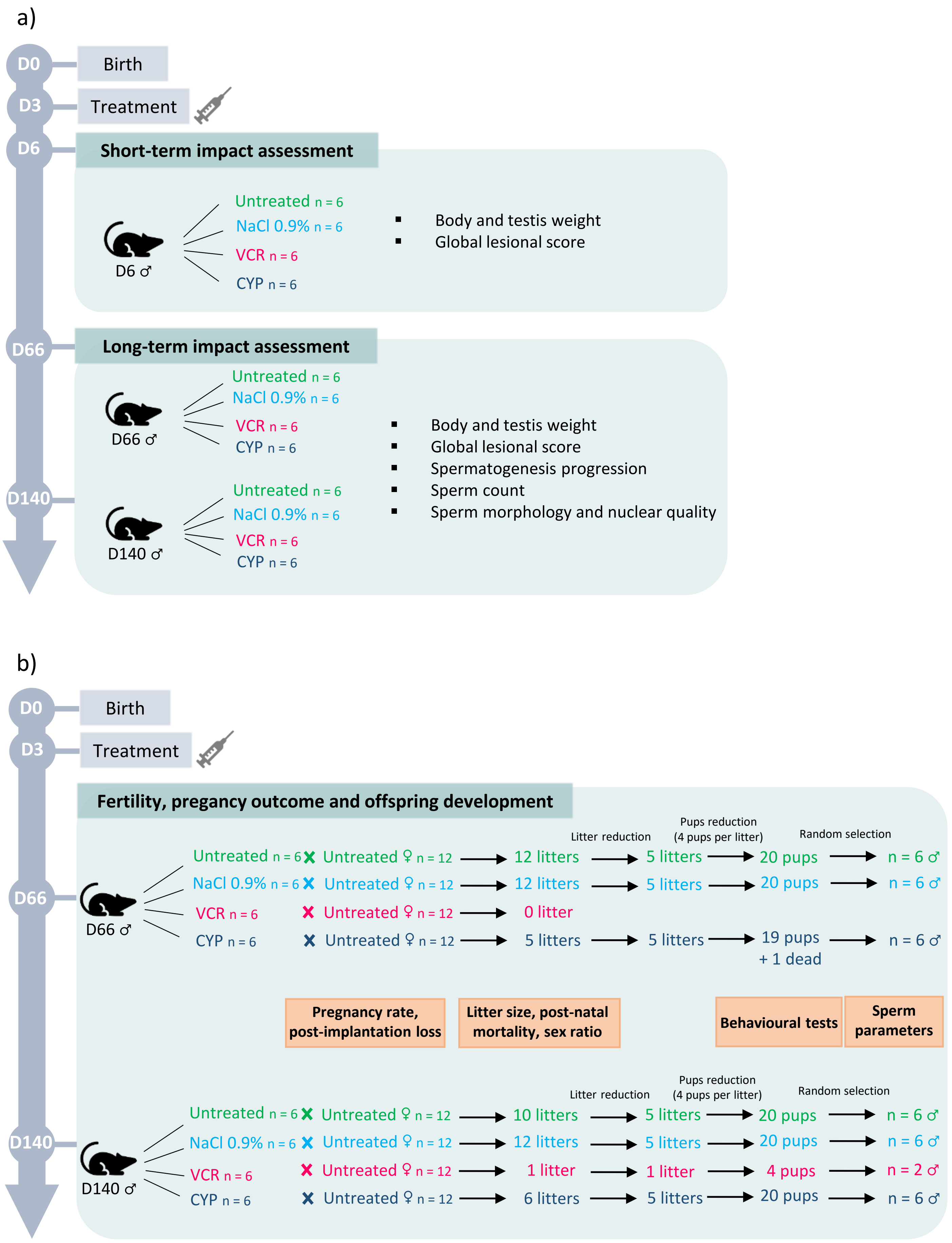

a)
D0
Birth
D3
Treatment
D6
Short-term impact assessment
Untreated n = 6
NaCl 0.9% n = 6
VCR n = 6
CYP n = 6
D6 ♂
Body and testis weight
Global lesional score
D66
Long-term impact assessment
Untreated n = 6
NaCl 0.9% n = 6
VCR n = 6
CYP n = 6
D66 ♂
Body and testis weight
Global lesional score
Spermatogenesis progression
Sperm count
Sperm morphology and nuclear quality
D140
Untreated n = 6
NaCl 0.9% n = 6
VCR n = 6
CYP n = 6
D140 ♂
b)
D0
Birth
D3
Treatment
Fertility, pregancy outcome and offspring development
Litter reduction
Pups reduction (4 pups per litter)
Random selection
D66
D66 ♂
Untreated n = 6 Untreated ♀ n = 12 12 litters 5 litters 20 pups n = 6 ♂
NaCl 0.9% n = 6 Untreated ♀ n = 12 12 litters 5 litters 20 pups n = 6 ♂
VCR n = 6 Untreated ♀ n = 12 0 litter
CYP n = 6 Untreated ♀ n = 12 5 litters 5 litters 19 pups + 1 dead n = 6 ♂
Pregnancy rate, post-implantation loss
Litter size, post-natal mortality, sex ratio
Behavioural tests
Sperm parameters
Litter reduction
Pups reduction (4 pups per litter)
Random selection
D140
D140 ♂
Untreated n = 6 Untreated ♀ n = 12 10 litters 5 litters 20 pups n = 6 ♂
NaCl 0.9% n = 6 Untreated ♀ n = 12 12 litters 5 litters 20 pups n = 6 ♂
VCR n = 6 Untreated ♀ n = 12 1 litter 1 litter 4 pups n = 2 ♂
CYP n = 6 Untreated ♀ n = 12 6 litters 5 litters 20 pups n = 6 ♂
